# Induction of decay accelerating factor and membrane cofactor protein by resveratrol attenuates complement deposition in human coronary artery endothelial cells

**DOI:** 10.1016/j.bbrep.2019.100652

**Published:** 2019-05-27

**Authors:** Maria G. Detsika, Eleni D. Myrtsi, Sofia D. Koulocheri, Serkos A. Haroutounian, Elias A. Lianos, Charis Roussos

**Affiliations:** a First Department of Critical Care Medicine and Pulmonary Services, Thorax Foundation, Research Center of Intensive Care and Emergency Thoracic Medicine, Evangelismos Hospital, School of Medicine, National and Kapodistrian University of Athens, Athens, Greece; b Department of Nutritional Physiology and Feeding, Agricultural University of Athens, Iera Odos 75, 11855, Athens, Greece

## Abstract

The involvement of complement activation in various forms of cardiovascular disease renders it an important factor for disease progression and therapeutic intervention. The protective effect of resveratrol against cardiovascular disease via moderate red wine consumption has been established but the exact mechanisms are still under investigation. The current study utilised human coronary artery endothelial cells (HCAECs) in order to assess the extent to which the protective effect of resveratrol, at concentrations present in red wine, can be attributed to the upregulation of complement regulatory proteins through heme-oxygenase (HO)-1 induction. Resveratrol at concentrations as low as 0.001 μΜ increased HO-1 expression as well as membrane cofactor protein (MCP, CD46) and decay-accelerating factor (DAF, CD55) expression with no-effect on CD59. Silencing of HO-1 expression by HO-1 siRNAs abrogated both DAF and MCP protein expression with no effect on CD59. Resveratrol-mediated induction of DAF and MCP reduced C3b deposition following incubation of HCAECs with 10% normal human serum or normal rat serum as a source of complement. Incubation of HCAECs, with either a DAF blocking antibody or following transfection with HO-1 siRNAs, in the presence of 10% normal rat serum increased C3b deposition, indicating that both DAF and HO-1 are required for C3b reduction. These observations support a novel mechanism for the protective effect of resveratrol against cardiovascular disease and confirm the important role of HO-1 in the regulation of the complement cascade.

## Highlights

• Resveratrol upregulated HO-1 protein in human coronary artery endothelial cells (HCAECs).
• Resveratrol upregulated complement control proteins DAF and MCP in HCAECs.
• HCAECs treatment with siHO-1 abolished HO-1 expression and reduced expression of both DAF and MCP.
• HCAECs resveratrol treatment in the presence of a source of complement reduced C3 deposition.

## Graphical abstract

**Figure:**
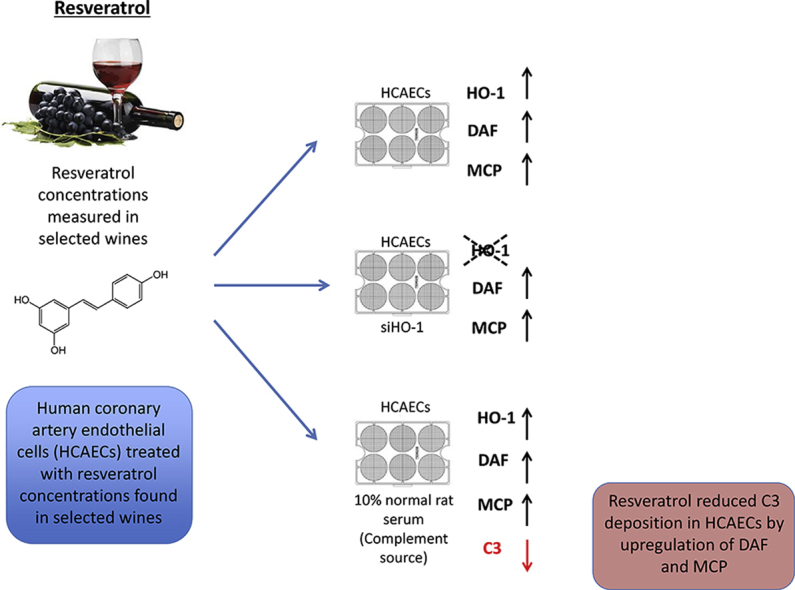

## 1 Introduction

Complement mediated inflammation has recently been gaining emphasis as an important contributor to various forms of cardiovascular disease including atherosclerosis, coronary heart disease and heart failure [1]. When activated, the complement cascade results in activation of the terminal pathway, which leads to lysis of cells or bacteria and subsequent release of inflammatory mediators [2]. Undesired or imbalanced activation of the complement system has been shown to lead to tissue damage and organ dysfunction. Complement activation is, therefore, under control by several proteins known as complement regulatory proteins. Key regulatory proteins are CD46 or membrane cofactor protein (MCP), CD55 or decay accelerating factor (DAF) and CD59 [3].

Moderate wine consumption is widely recognised as protective against cardiovascular disease and is mainly attributed to phenolic stilbenes, exemplified by *trans-*resveratrol [3,5,4′-trihydroxy-*trans*-stilbene,(E)-5-(4-hydroxystyryl)benzene-1,3-diol], a naturally occurring phytoalexin widely known as resveratrol. Initially, the demonstration of a reverse relationship between wine consumption and cardiovascular disease [4], also known as the ‘French paradox’, was mainly attributed to the various phenolic compounds of the stilbene family contained in wine, especially red wine. In 1992 [5] resveratrol was identified as a key red wine bioactive polyphenol and, based on extensive research, was implicated in conferring the protective effect of red wine against cardiovascular disease. Other health promoting effects linked to resveratrol include increased longevity and attenuation of diet-induced metabolic syndrome and diabetes [[6], [7], [8]].

The mechanisms underlying the protective effects of resveratrol in cardiovascular diseases are still under investigation and include the induction of the cytoprotective enzyme heme oxygenase (HO)-1 [9]. HO-1 is the rate-limiting enzyme of heme degradation to biliverdin, carbon monoxide, iron, and bilirubin. Cytoprotective effects of HO-1 induction have been attributed to anti-inflammatory, anti-apoptotic, anti-proliferative, and antioxidant effects of these heme degradation products [10]. Moreover, HO-1 induction was shown to enhance vascular endothelial resistance to complement-mediated injury [11], and our previous studies demonstrated that in the renal glomerular microvasculature HO-1 upregulates DAF and reduces C3 deposition [12].

Herein we have hypothesized that resveratrol, at concentrations contained in wine, increase expression of complement regulatory proteins and this effect is mediated via HO-1 induction. To test this hypothesis, we determined resveratrol concentrations in randomly selected wines from specific grape varieties cultivated in Greece and assessed the effect of equivalent concentrations of chemically pure resveratrol on complement regulatory proteins expression and complement deposition in human coronary artery endothelial cells (HCAECs).

## 2 Materials and methods

### 2.1 Reagents

Anti–HO–1 antibody (catalog number ADI-SPA-896) was purchased from Enzo Life Sciences (New York, USA). Anti-DAF (clone 1C6, catalog number HM2280), anti-MCP (clone M177, catalog number HM2103) anti-CD59 (clone MEM-43, catalog number HM2120), anti-rat C3/C3b (clone 2B10B9B2; catalog number HM3031) and anti-human C3/C3b (clone 755, catalog number: HM2072) antibodies were purchased from Hycult (Uden, Netherlands). Anti-β-actin antibody (catalog number 4967) was purchased from Cell Signaling (Danvers, MA). *Trans*-resveratrol (99%) and *trans*-piceid (95%) standards were purchased from Sigma-Aldrich. Water, acetonitrile and methanol were obtained from Fisher Chemicals. LC-MS grade solvents and analytical grade ethanol were from Merck. Commercially available wines from the island of Santorini were randomly selected from local wine producers. Winery of origin, name, variety, type and vintage year of wines in which *trans*-resveratrol and *trans*-piceid concentrateons were measured are listed in Table 1.

**Table 1:** White and red wines in which resveratrol and piceid levels were determined.

Sample	Winery	Name of Wine	Variety	Wine Type	VintageYear
1	Artemis Karamolegos	Nykteri	Assyrtiko, Athyri, Aidani	White Dry	2015
2	Artemis Karamolegos	Assyrtiko Santorini	Assyrtiko	White Dry	2016
3	Artemis Karamolegos	Aidani	Aidani	White Dry	2016
4	Canava Roussos	Nykteri	Assyrtiko	White Dry	2016
5	Canava Roussos	Caldera	Assyrtiko, Mandilaria	Red Dry	2007
6	Artemis Karamolegos	Mavrotragano	Mavrotragano	Red Dry	2016
7	Artemis Karamolegos	TerraNera	Mandilaria	Red Dry	2016
8	Canava Roussos	Mavrathiro	Assyrtiko, Mandilaria, Mavrathiro	Red Sweet	nR^a^
9	Artemis Karamolegos	TerraNera	Assyrtiko, Mandilaria	Rose Dry	2016

a nR, not Reported.

### 2.2 UPLC-MS/MS analysis of resveratrol and piceid levels in wine varieties

Stock solutions prepared consisted of methanolic solutions (100 μg/mL) of *trans*-resveratrol and *trans*-piceid (resveratrol 3-O-beta-D-glucoside). To avoid decomposition, all stock solutions were maintained at -4 °C in the dark. Stock solutions were diluted with water-acetonitrile (90:10) to achieve concentrations ranging from 0.219 to 43.813 μM for *trans*-resveratrol and 0.128–25.616 μM for *trans*-piceid. These were utilised for the construction of calibration curves immediately prior the analyses. For each wine sample, duplicate analyses were carried out by direct injection into UPLC-MS/MS immediately after the bottle opening. All experimental procedures were performed in the absence of direct sunlight and below 35 °C to avoid polyphenol isomerization.

UPLC-MS/MS analyses were carried out using an Accela Ultra High-Performance Liquid Chromatography system (Thermo Fisher Scientific, Waltham, MA, USA) coupled with a TSQ Quantum Access triple quadrupole mass spectrometer operated in multiple reaction monitoring mode and equipped with an autosampler (Thermo Fischer Scientific, San Jose, CA, USA). Mass spectrometric analysis was conducted using heated electrospray ionization (HESI), which was operated in two complementary modes (positive and negative). Additionally, a selected reaction monitoring mode was used to confirm the presence of analytes. The ion source and vacuum parameters of mass spectrometer were optimized to be applicable to all analytes. These parameters, as well as determination of molecular ion transitions and the collision activated ionization for the target analytes were obtained by direct infusion in full scan mode of their standard solutions displaying concentration 4.40 μM for *trans*-resveratrol and 2.56 μM for *trans*-piceid. The ESI ionization source was adjusted in negative polarity because of the sensitivity for the two target compounds in this ion mode. The spray voltage was set at 2700 V, sheath gas (nitrogen) and auxiliary gas (argon) pressures were set at 25 and 10 Arb, respectively. Capillary temperature was set at 320 °C and collision pressure at 1.5 mTorr. The monitoring ion transitions were *m*/*z* 227.9 > 186.5 (collision energy 22 eV) and 227.9 > 143.9 (collision energy 29 eV) for *trans*-resveratrol and 389.8 > 227.6, 389.8 > 186.0 and *m*/*z* 389.8 > 143.6 for the *trans*-piceid with collision energy 26, 43 and 54 eV, respectively.

Polyphenols were separated on a Hypersil Gold 100 × 2.1 cm chromatographic column (Thermo Fischer Scientific, San Jos, CA), using a mobile phase consisting of water (A) and acetonitrile (B) with the addition of formic acid (0.1%) in each solvent. The gradient program was: 0.0–1.0 min: 10% B, 1.0–12.0 min from 10% B to 100%, and 12.1–14.0 min 10% B tore-equilibrate the column, with 300 μL/min flow rate, and injection volume 10 μL and maintaining the column temperature at 35 °C. The retention times of *trans*-resveratrol and *trans*-piceid were 4.17 and 5.30 min, respectively.

### 2.3 Culture and resveratrol treatment of HCAECs

HCAECs (Sigma) were routinely maintained in Meso Endo Growth media (Cell Applications Inc.) at 37 °C in 5% CO_2._ Experiments were performed between passages 4–8. Treatments were performed when cells reached 80% confluency. Resveratrol solution was prepared fresh each time in dimethylsulphoxide (DMSO). Cells were treated with resveratrol concentrations (0.001–0.1 μM) for 18 h. For the time course experiment, cells were treated with resveratrol (0.001 μM) for specific time points (1 h and 3 h). Upon completion of treatments, cells were harvested for protein extraction using lysis buffer and concentration was determined as previously described [12].

### 2.4 Cell transfection and treatments

Cells were transfected with oligonucleotides (100 nM) for HO-1 silencing or negative control siRNA (Intergrated DNA Technologies, IDT). Briefly, one day before transfection cells were plated in Meso Endo medium so that cells would be at 50% confluency at the time of transfection. The following day cells were transfected with siRNAs in unsupplemented medium and Oligofectamine (Invitrogen) for 48 h. Following transfection, cells were treated with resveratrol and further incubated for 18 h.

### 2.5 Western blotting

Protein lysates were resolved by SDS-PAGE as previously described [13]. Primary antibody dilutions were: 1:2000 for HO-1 antibody, 1:50 for anti-DAF, anti-MCP, anti-human and anti-rat C3b antibodies, 1:100 for anti-CD59 antibody and 1:5000 for anti-β-actin. Secondary antibodies (Cell Signaling) were diluted at 1:2000.

### 2.6 Reverse transcription (RT) and real-time PCR amplification

Cells were harvested for RNA extraction in Trizol as previously described [12]. Cycling conditions included the following steps: 50 °C for 5 min, 95 °C for 10 min, and 45 cycles of 5 min at 95 °C and 1 min at 60 °C in a BioRad CFX Connect thermal cycler. Levels of HO-1 and DAF mRNA were normalised to glyceraldehyde 3-phosphate dehydrogenase GAPDH. Reactions were carried out in triplicate and results were analysed by the ΔΔCT method.

### 2.7 Statistical analyses

Values are expressed as mean ± standard error of the mean (SEM). Statistical analyses were performed with either *t*-test, where applicable, or analysis of variance (ANOVA). When significant, post hoc analysis was performed with either a Turkey or the least significant difference (LSD) test. A p value < 0.05 was chosen as statistically significant.

## 3 Results

### 3.1 HO-1 and DAF expression induction in HCAECs by resveratrol

Resveratrol concentrations ranging from 0.001 to 0.1 μM were chosen for an initial dose response experiment in HCAECs based on resveratrol (*trans*-resveratrol) and piceid (*trans*-piceid) concentration measurements obtained by UPLC-MS/MS in wine samples tested (Table 2). Piceid is a glycosylated derivative of resveratrol, which has been shown to be present in larger amounts compared to resveratrol in some varieties of wine [14]. Upon hydrolysis of this glycoside in the liver and intestine, resveratrol is released thus adding to the total amount of resveratrol uptake from diet [15]. Considering that piceid is readily hydrolysed into resveratrol, total levels of resveratrol (the sum of *trans*-resveratrol and *trans*-piceid concentrations) were determined in all varieties tested (Table 2). Total resveratrol concentrations ranged between 0.102 and 0.238 μM for white varieties and between 0.342 and 6.482 μM for red varieties (Table 2). If we assume consumption of a glass of white wine (300 ml) containing the lowest resveratrol concentration observed (0.102 μM) and a resveratrol distribution in 5 L of blood, the final resveratrol concentration in circulation is estimated to be 0.006 μM. We, therefore, chose 0.001 μM as the lowest concentration for testing in the dose response experiment.

**Table 2:** *Trans*-resveratrol and *trans*-piceid concentrations in wine-samples.

Sample	*trans*-resveratrol (μM)	*trans*-piceid (μM)	Total (μM)^c^
1	nD^a^	0.238	0.238
2	nD	0.179	0.179
3	nD	0.102	0.102
4	nD	0.223	0.223
5	nD	0.202	0.202
6	4.280	2.282	6.562
7	1.700	1.578	3.278
8	0.342	nD	0.342
9	<DL^b^	0.738	0.738

a nD, not Detected.

b < DL, below Detection Limit.

c Sum of *trans*-resveratrol and *trans*-piceid concentration.

**Fig. 1:**
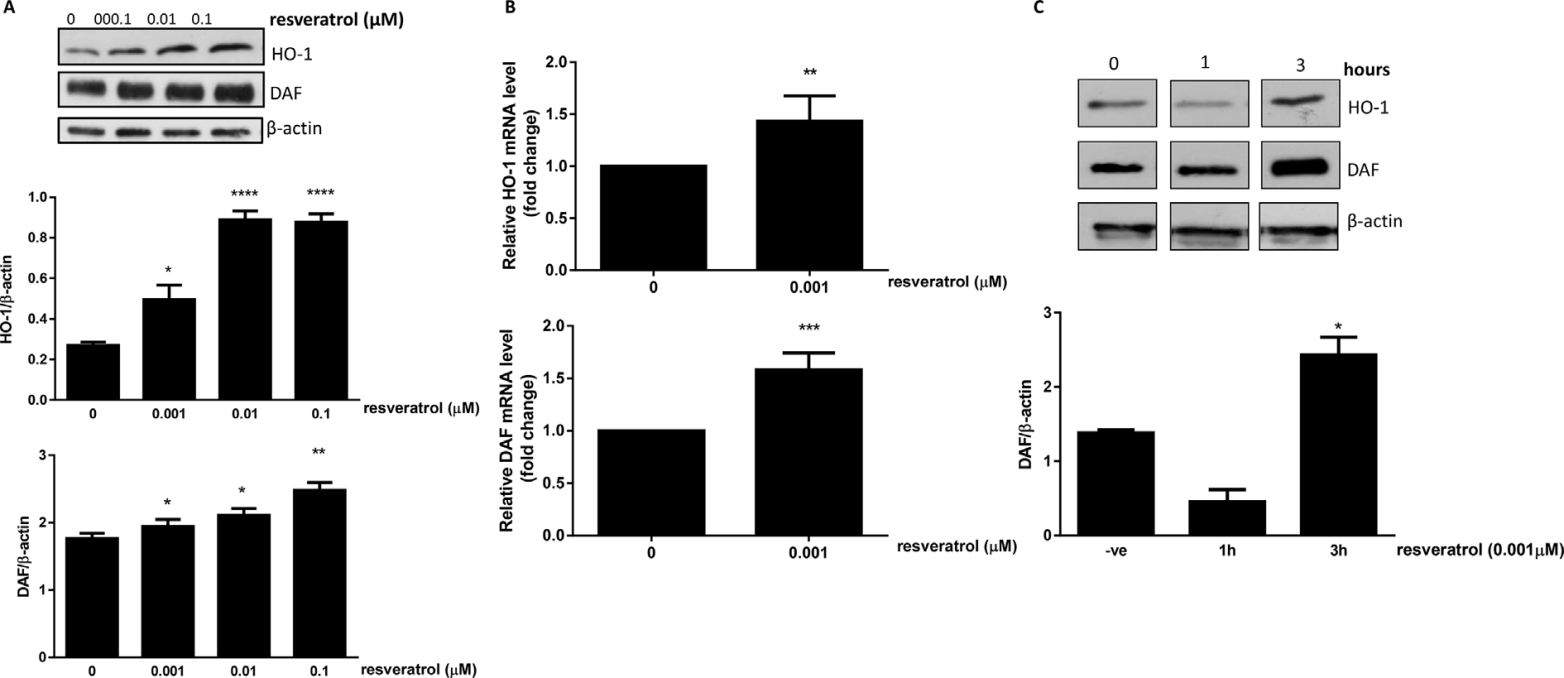
**Dose and time dependent induction of HO-1 and DAF protein and mRNA levels in response to resveratrol.** HCAECs were incubated with resveratrol (0.001–0.1 μΜ) for 18 h. HO-1 and DAF proteins were analysed by immunoblotting and quantified by densitometry (A). Data are presented as mean ± SEM (n = 3). β-actin was used as a loading control. *p < 0.05, **p < 0.01, ***p < 0.001 and ****p < 0.0001 vs 0 resveratrol. (B) HO-1 and DAF mRNA levels were determined by Real-time PCR. Values are expressed as fold change compared to control set to 1. (C) HCAECs were incubated with resveratrol (0.001 μΜ) for 1 and 3 h. HO-1 and DAF proteins were analysed by immunoblotting. A representative Western blot is shown (n = 3). DAF protein was quantified by densitometry. Data are presented as mean ± SEM (n = 3). β-actin was used as a loading control. *p < 0.05.

Treatment of HCAECs with resveratrol (0.001–01 μM) induced both HO-1 and DAF protein expression (Fig. 1A). HO-1 and DAF protein increased significantly at concentration as low as 0.001 μM (Fig. 1A). The same concentration also increased HO-1 and DAF mRNA levels (Fig. 1B) and was chosen for subsequent experiments. The increase in HO-1 and DAF protein was observed following a 3-h incubation with resveratrol (0.001 μM**)** (Fig. 1C).

### 3.2 HO-1 mediates DAF induction in response to resveratrol

**Fig. 2:**
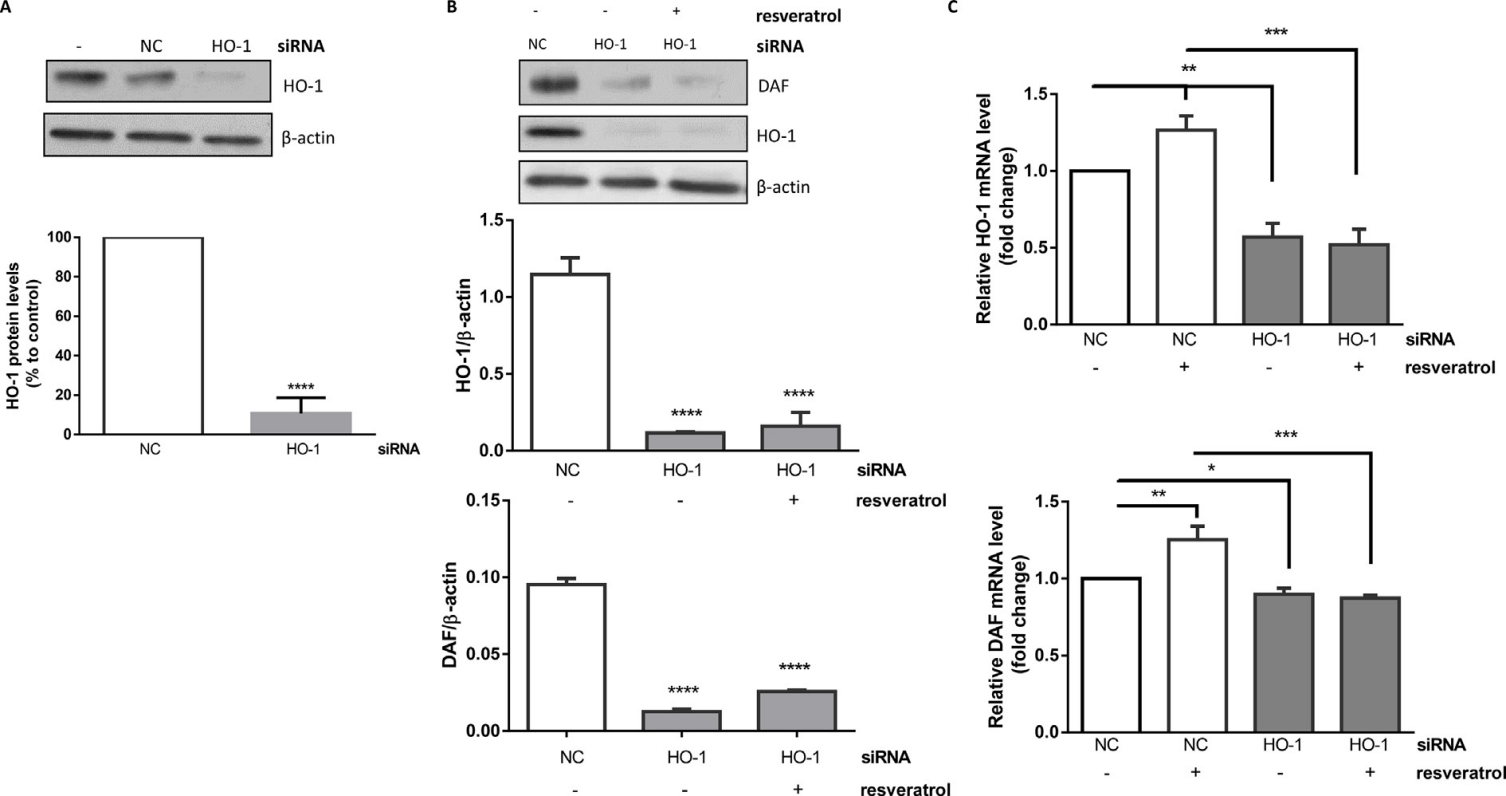
**Effect of HO-1 silencing on basal and resveratrol-mediated DAF induction.** (A) HCAECs were treated with 100 nM negative control (NC) siRNA or HO-1 siRNA for 48 h. (B) HCAECs were treated with 100 nM NC siRNA or HO-1 siRNA for 48 h prior to resveratrol (0.001 μΜ) treatment for 18 h. HO-1 and DAF protein and mRNA levels were analysed by immunoblotting (B) or real-time PCR respectively (C). HO-1 and DAF proteins were quantified by densitometry. Data are presented as mean ± SEM (n = 3). β-actin was used as a loading control. *p < 0.05, ****p < 0.0001 vs NC. Relative mRNA levels are expressed as fold change compared to control set to 1 and mean ± SEM (n = 3). *p < 0.05, **p < 0.01, ***p < 0.001.

Validation of HO-1 siRNAs in HCAECs revealed ∼90% reduction of HO-1 protein compared to negative control (Fig. 2A). To determine whether upregulation of DAF by resveratrol requires HO-1, HCAECs were treated with negative control (NC) siRNA or HO-1 siRNAs in the presence or absence of resveratrol. HO-1 silencing markedly reduced DAF protein and mRNA expression (Fig. 2B and C) indicating that HO-1 regulates DAF. Resveratrol treatment (0.001 μM) of HCAECs following transfection with HO-1 siRNA did not reverse the decrease in DAF protein indicating that HO-1 is essential for resveratrol-mediated DAF induction (Fig. 2B and C).

### 3.3 Effect of resveratrol on MCP and CD59

**Fig. 3:**
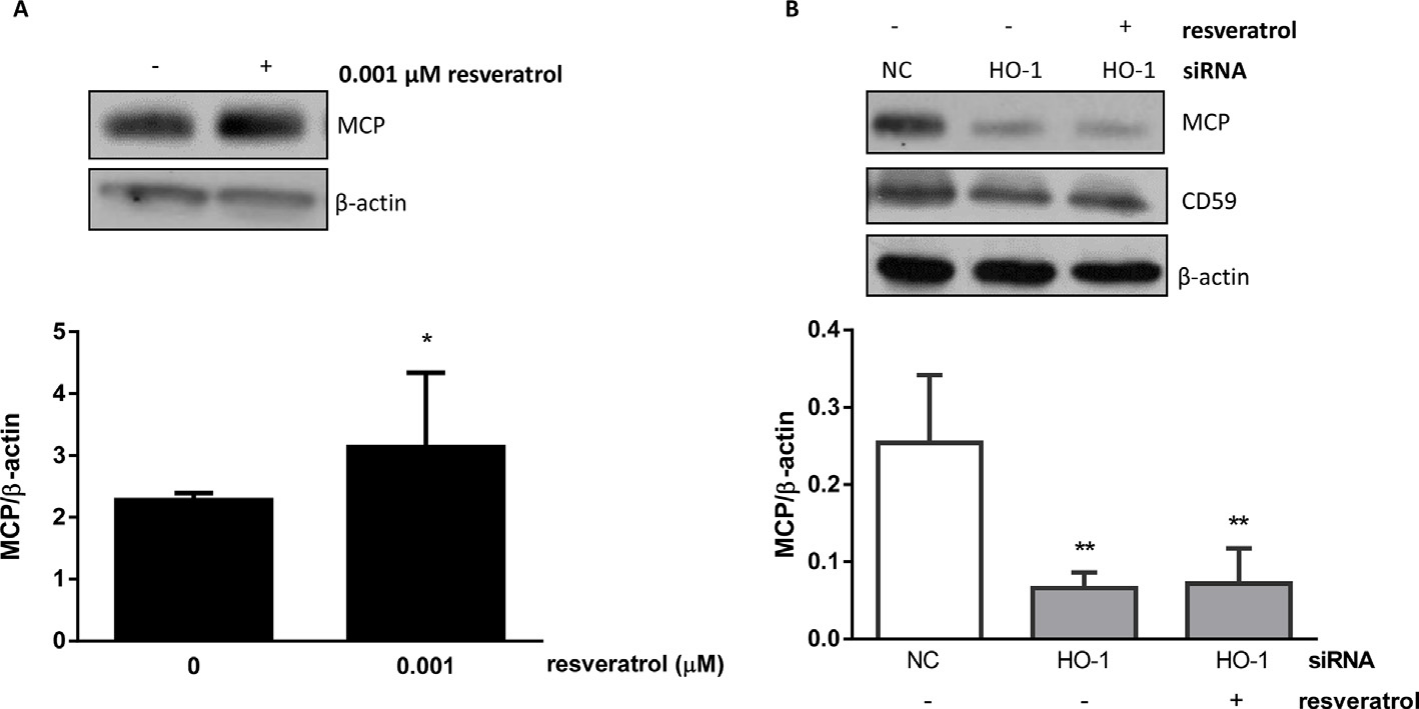
**Resveratrol increases MCP expression via a HO-1 dependent mechanism.** (A) HCAECs were treated with resveratrol (0.001 μΜ) for 18 h and (B) HCAECs were transfected with 100 nM negative control (NC) or HO-1 siRNA for 48 h prior to resveratrol (0.001 μΜ) treatment for 18 h. MCP and CD59 proteins were analysed by immunoblotting and quantified by densitometry. Data are expressed as mean ± SEM (n = 3). β-actin was used as a loading control. *p < 0.05 vs no resveratrol, **p < 0.01 vs NC.

As shown in Fig. 3A HCAEC treatment with resveratrol (0.001 μM) significantly increased MCP protein levels. There was no effect on CD59 (not shown). HO-1 silencing reduced MCP protein in the presence or absence of resveratrol (Fig. 3B) indicating that HO-1 also regulates resveratrol-mediated MCP expression (Fig. 3B).

### 3.4 Resveratrol reduces C3 deposition

**Fig. 4:**
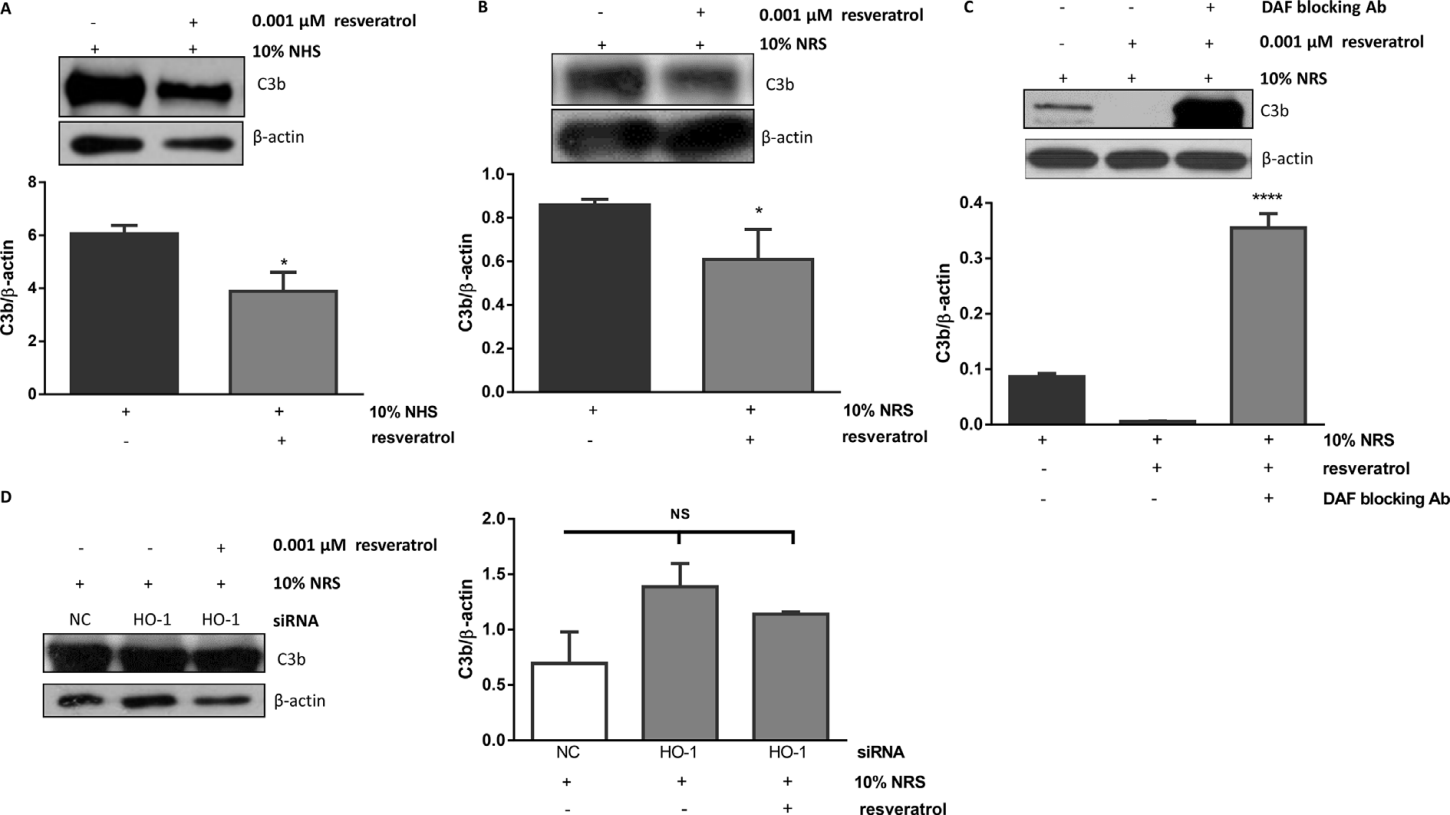
**DAF upregulation by resveratrol reduces C3 deposition.** (A) HCAECs were incubated in the presence of 10% normal human (NHS) or (B) normal rat serum (NRS, 10%) and treated with resveratrol (0.001 μΜ). (C) HCAECs were pretreated with a DAF blocking antibody for 15 min before resveratrol (0.001 μΜ) treatment in the presence of 10% NRS. (D) HCAECs were transfected with 100 nM negative control (NC) or HO-1 siRNA for 48 h prior to resveratrol treatment (0.001 μΜ) in the presence of 10% NRS. C3 levels were analysed by immunoblotting and quantified by densitometry. Data are expressed as mean ± SEM (n = 3). β-actin was used as a loading control. *p < 0.05 vs no resveratrol, ****p < 0.0001 vs 10% NRS only, NS; non-significant.

To determine whether DAF upregulation in response to resveratrol is sufficient to reduce C3b deposition, an *in-vitro* assay we described previously [12] was utilised. Specifically, HCAECs were incubated in the presence of a complement source (10% normal human (NHS) or rat serum, NRS) in order to trigger spontaneous complement activation and C3b deposition in the presence and absence of resveratrol (0.001 μM). The extent to which DAF induction decreased C3b deposition was then assessed by Western blot analysis. Resveratrol significantly reduced C3b deposition in the presence of NHS (Fig. 4A) or NRS (Fig. 4B). In the presence of a DAF blocking antibody, C3b levels were increased indicating that the reduction of C3b observed was dependent on DAF induction (Fig. 4C). Incubation of HCAECs with 10% NRS in the presence or absence of resveratrol following HO-1 silencing also increased C3b deposition, although non-significantly, indicating that both HO-1 and DAF are required for the reduction in C3b deposition (Fig. 4D).

## 4 Discussion

The role of complement activation in cardiovascular disease prediction, emergence and progression has been demonstrated by numerous studies rendering it an important pathogenetic factor in cardiovascular disease. Increased C3 levels have been reported as a prognostic marker of future cardiovascular events in both men and women [16,17] and were associated with an increased risk for coronary heart disease [18]. These findings were further supported by a recent study demonstrating a strong correlation between increased C3, C4 and C3ades-Arg with adipose tissue volume and other well-established coronary heart disease factors [19]. Complement activation is also involved in atherosclerosis, with studies showing elevated deposition of C3 in atherosclerotic lesions as well as presence of C5b-9 and complement regulatory proteins within atherosclerotic plaques [20,21] suggesting that full complement activation takes place [22].

Significantly, C3 and terminal complement component deposition is also found in coronary arteries of patients with systemic inflammatory diseases including lupus erythematosus and rheumatoid arthritis, and this was proposed as an underlying mechanism of accelerated coronary artery disease known to occur in these patients [23,24]. Spontaneous complement activation and formation of C3 fragments in human endothelium can also occur via the C3 “tick-over” effect occurring in the alternative complement activation pathway or C1 “tick-over” in the classical pathway [25].

The present study demonstrates that upregulation of DAF and MCP in HCAECs is a potential mechanism underlying known protective effects of resveratrol against cardiovascular disease. Both DAF and MCP induction in response to resveratrol were HO-1 dependent as HO-1 knock-down abrogated their induction, and this effect could not be reversed by resveratrol (Fig. 2, Fig. 3). In a previous study, it was shown that adenoviral overexpression of HO-1 in human umbilical vein endothelial cells resulted in DAF but not MCP or CD59 over-expression [11]. In HCAECs employed in the present study, resveratrol-mediated HO-1 induction increased both DAF and MCP but not CD59 expression.

MCP is a transmembrane regulatory protein, which acts as a cofactor for factor I-mediated enzymatic cleavage of the C3 and C5 convertase enzyme subunits C4b and C3b, respectively, and has a complementary regulatory activity to DAF. While both MCP and DAF act at the level of the C3 convertase, DAF has decay-accelerating activity but, contrary to MCP, it has no co-factor activity [3]. DAF is initially synthesized as a precursor (46 kDa) molecule, which gives rise to mature DAF on the cell surface as a 70–80 kDa protein [26]. It binds complement components C3b and C4b thereby preventing assembly and accelerating decay of C3 and C5 convertases, which serve to amplify the complement activation cascades [27]. CD59 inhibits formation of the membrane attack complex (MAC) after the stage of C5b-7 insertion into cell membranes.

In contrast to DAF and CD59, which are tethered to cell membrane via a glycosylphosphatidylinositol (gpi) anchor, MCP is tethered via a transmembrane domain. However, both DAF and MCP are composed of four short consensus repeat regions (SCRs) and a Ser/Thr rich region, in the case of DAF, or a Ser/Thr/Pro rich region, in the case of MCP, which attach on the anchor or the transmembrane domain, respectively [27]. Homology between DAF and MCP SCRs has previously been identified [28]. The fact that both MCP and DAF expression was decreased by HO-1 silencing and that, in contrast to CD59, both were induced by resveratrol, suggests that HO-1 could be regulating MCP and DAF at the level of the SCR sequence, which is absent from CD59 protein.

Previous studies on resveratrol effects on outcomes of microvascular diseases in which complement activation has been implicated as a contributory mechanism of injury have been performed both in human and animal models. In a previous study, pretreatment of human neutrophils with resveratrol, was shown to significantly block oxidative burst, leukocyte migration, degranulation, and inflammatory cytokine production [29]. Finally, in a murine model of complement-dependent injury of the glomerular microvasculature resembling human membranous nephropathy, daily subcutaneous injections of resveratrol upregulated HO-1 and significantly reduced glomerular C3 deposition and production of reactive oxygen species [30].

In our study, resveratrol increased HCAEC HO-1 and DAF expression at concentrations (0.001 μM) that were far lower than those measured in randomly selected wines (Table 1) or those reported in previous studies [31]. Moreover, DAF induction in response to 0.001 μM resveratrol was sufficient to decrease C3b deposition (Fig. 4A and B) and this effect was reversed in the presence of a DAF blocking antibody (Fig. 4C). HO-1 knock-down in the presence or absence of resveratrol abrogated DAF expression and increased C3b deposition albeit non-significantly (Fig. 4D). Taken together, these observations indicate that HO-1 is required for resveratrol-mediated DAF and MCP induction and reduced C3b deposition in HCAECs. Furthermore, the ability of resveratrol to reduce C3b levels at such low concentrations increases its importance in cardiovascular disease and atherosclerosis.

The mechanism underlying resveratrol-mediated HO-1 and DAF induction remains to be elucidated. In a microarray study, Nicholson and co-workers assessed effects of dietary polyphenols on human vascular endothelial cell gene expression. Resveratrol treatment strongly modulated gene expression, leading to significant (more than two-fold) downregulation of 363 genes and upregulation of 233 genes of the 10,000 genes present on the microarray [32]. A likely mechanism of resveratrol-mediated DAF induction is increased activity AMP-activated protein kinase (AMPK) [33] as DAF promoter contains a cAMP response element [34].

Resveratrol administration in animal models of cardiovascular injury has been shown to ameliorate extent of injury and was mainly attributed to reduction of oxidative stress [9]. Furthermore, in a model of antibody-mediated injury of epithelial cells of the renal glomerular microvasculature, resveratrol administration attenuated complement-mediated injury [30]. However, the role of complement following resveratrol administration, has not been investigated. The current study provides a novel finding which can be further investigated in animal models of coronary heart disease in order to dissect the role of complement controllers in reducing extent of injury.

In summary the current study describes a novel mechanism that could explain the protective effect of resveratrol against complement-mediated cardiovascular injury and introduces a novel therapeutic role of resveratrol against complement mediated diseases. The mechanism involves upregulation of complement activation controllers DAF and MCP via HO-1 induction and this confirms the important role of HO-1 in regulating the complement cascade.

## Conflicts of interest

All authors declare no conflict of interest.
